# F. H. Clark’s Patent Improved Method of Inserting Pivot Teeth

**Published:** 1849-04

**Authors:** 


From the Dental Recorder.

### F. H. CLARK'S PATENT IMPROVED METHOD OF INSERTING PIVOT TEETH.

THIS improvement consists of a cylindrical lining, of a size to fit the opening usually made in the root of a tooth for the reception of a wooden pivot. This cylinder has a bottom of a spherical form, with a hole in its centre, through which a screw passes into the natural canal of the root, which is carefully prepared for its reception by "tapping," &c. The cylinder has likewise a phlange or collar around the other end, to enable the dentist to fill with gold any cavity which may have formed in the root, (however large it may be,) if its edge is not obliterated. The inside of this cylinder has, near its bottom, an elevation or bar soldered across, to serve as a catch for an elastic metalic pivot.

The screw which passes through the bottom of the cylinder, and holds it in its place, is usually perforated through its length, for the purpose of allowing the escape of pus in case of disease of the alveolus or periosteum. The propriety of allowing this matter to escape through the root by means of its natural canal, is an undecided question; the perforated screw, however, will enable the dentist to decide for himself, as he can close and open it at pleasure.

The advantages claimed for this mode of inserting pivot teeth, are as follows; First, the root being lined with gold, and its end being filled or covered with the same metal, it is protected from further decay. Second, no impure matter finds a permanent lodgment between the root and tooth, as the tooth is made removable at the pleasure of the wearer, for the purpose of cleansing. This, it is believed, will prevent the offensive odor inseparable from the usual method of inserting teeth by means of the wooden pivot. Third, it is confidently asserted that a root thus protected will last many years longer; and if this is true, an early resort to a gold plate is avoided, and the destruction of the teeth to which it is attached prevented.

The above differs essentially so little from the plan recommended by us in our lectures to the dental class, that we give it in connection with the above.

"In pivoting teeth when the roots are not perfectly sound, and the gums show no particular indications of disease, the ordinary method of inserting with wood, or wood and wire through its centre, will not hold the teeth with that firmness which is requisite, nor preserve the roots from that rapid softening which is so often met with, and which renders these teeth so useless To obviate this difficulty and preserve the roots, as well as render these teeth more secure, we formerly were in the habit of first inserting our pivot into the cavity in the fang, then plugging compactly around this with foil, leaving the wood slightly enlarged at the end, so as to, in part at least, retain this filling. Then adjust the tooth, sometimes making the wood and foil to merely plug up the root, leaving the base, on which the artificial tooth sets, smooth and solid, representing a healthy, sound root, with a hole pierced through the centre of the wood, to represent the dental foramen, and into this force a metalic pivot, which has been adjusted to an artificial crown. The necessity, however, of at times leaving an outlet for the escape of matter, and which was not very easily done when this plan was adopted, led us to an improvement which we consider far preferable. This consists in the formation of a gold tube or cylinder, the length of which you desire your pivot. The chamber of this tube is divided by a plate of the same material, first soldered to its place before the tube is closed, and which, however, should not divide the tube in the centre, but leave on one side a space twice as large as on the other. The larger space is for the reception of the pivot, the smaller for the escape of matter. On one end of this tube is soldered a head or phlange—this is for the better retention of the gold filling, which is to be introduced after the insertion of the tube. On the other end of this cylinder is cut a screw—this is made of the proper size to suit the cavity already formed in the fang, and which has been threaded or cut with a screw for the reception of your tube. This should be inserted in such a manner that the smaller opening in your cylinder should be at the palital side of the fang, and before the insertion of your tooth, care should be taken to see that the dental foramen is open to the apex of the fang, and has access to the smaller opening through which the matter is to pass; from this opening in the tube, to the palital edge of the fang, a small groove is cut, which carries the matter into the mouth. The decayed portion of the root having been removed, and, where admissible, one or two circular grooves cut in the flaring portion of the cavity, the tube is screwed in, and a solid compact plugging of gold made to fill up the entire root not filled by the tube; after which the base is leveled with a file, and your tooth, which is prepared, with a metalic pivot, (which is accurately adapted to the larger opening of the cylinder,) is then inserted—this may be so adjusted as that it can be removed or not as may seem best.

"This method, so well adapted for these cases, has also led me to adopt the same, or nearly the same, where it is not necessary to leave an opening for egress of matter. In the latter case making the tube without the partition, and generally some smaller than where two openings are necessary. After or before the tube is screwed into the root, enlarge the dental foramen some distance up the same size of the cavity in the tube, and then make the pivot long enough to pierce this as far as possible, thus securing a firm and solid tooth. In the latter operation, the tube is inserted, and made as in the former, excepting that there is no partition in its cavity, and the pivot may pass entirely through this into the dental foramen."

These remarks, taken from our lecture to the class, will give, as we think, a tolerable idea of our plan; and we would here remark, that we have on several occasions used these tubes for the insertion of plate teeth, in one instance using two good roots for the retention of seven teeth. The plate is very narrow, made double, is removed every day, cleansed and replaced, by the lady who wears them, and has been in use now three to four years, and, we must say, look as well and subserve as good a purpose as the same number of teeth ever inserted by us either before or since. The roots appear to be still sound and healthy, and we are sure the lady would not part with them for any teeth that might be clasped to her other teeth, which are quite good. We wish, however, not to be understood as recommending the general adoption of this plan; for we know that is generally not only advisable, but necessary, to remove these roots from the mouth; and so it is the case with those roots which have abscesses formed at their apex. It shows, however, the resort of the profession, and, as we believe, sometimes justifiable, because inflicting on our patient a lesser evil than the adaptation of clasps to teeth; the mouth should, however, always be properly prepared and put in a healthy condition. We hold that no mouth in a diseased condition is fit for the reception of artificial teeth, and are not at all surprised at seeing so many pivot teeth fail in two or three years; it is only a matter of astonishment that they can often be worn at all. The gums are often spongy and inflamed when these teeth are inserted; many decayed teeth and roots are suffered to remain in the mouth, which, of themselves, cause continued suffering. In this condition of things, artificial teeth only increase the general irritation.—[CIN. ED.

